# Recombinant human interferon‐α14 for the treatment of canine allergic pruritic disease in eight dogs

**DOI:** 10.1002/vro2.6

**Published:** 2021-05-02

**Authors:** Breno C. B. Beirão, Aline C. Taraciuk, Carolina Trentin, Max Ingberman, Luiz F. Caron, Chris McKenzie, William H. Stimson

**Affiliations:** ^1^ Imunova Análises Biológicas LTDA Curitiba Brazil; ^2^ Departamento de Patologia Básica Universidade Federal do Paraná Curitiba Brazil; ^3^ Veterinary Consultant, Avenida Nossa Senhora de Lourdes,63 Curitiba Brazil; ^4^ ILC Therapeutics Ltd. Biocity Scotland Lanarkshire UK; ^5^ Immunology Department Strathclyde University Glasgow Scotland UK

**Keywords:** allergic, atopic dermatitis, dog, interferon, pruritic diseases

## Abstract

**Background:**

Allergic pruritic diseases are increasingly common in dogs. This group of conditions hampers life quality as pruritus progressively interferes with normal behaviours. Therefore, new treatment modalities for canine allergic pruritic diseases are necessary. While novel drugs have recently reached the market, there is still the need for other therapeutic approaches. Some dogs are refractory even to the newer compounds, and cost is also an important issue for these. Older therapeutic modalities are only moderately successful or have considerable secondary effects, as is the case with glucocorticoids.

**Objectives:**

Report on the use of recombinant human interferon‐α14 (rhIFN‐α14) for the treatment of canine allergic pruritus. Following the experience with a similar compound in the Japanese market, it was expected that rhIFN‐α14 could alter the Th1/Th2 disbalance that drives these diseases.

**Methods:**

Here, we present an uncontrolled trial in which eight dogs with clinical diagnosis of allergic pruritus were treated with rhIFN‐α14, either orally or via subcutaneous injections. Skin condition, microbiota and anti‐interferon antibody levels were assessed.

**Results:**

The parenteral use of interferon induced hypersensitivity in two of the three dogs in which it was used. The oral administration was consistently safe and could reduce signs of the allergic condition in three of the five treated animals. Treatment also altered the skin microbiota, as verified by next‐generation sequencing.

**Conclusion:**

The present results indicate that rhIFN‐α14 is a viable candidate for the treatment of canine allergic pruritus. Future controlled studies are needed, and the oral route is indicated for further trials.

## INTRODUCTION

Canine allergic pruritic diseases are a common condition in dogs, characterised by dermatosis with intense pruritus and inflammation. Atopic dermatitis (AD) is the primary variety of allergic skin conditions.^1^ The most common clinical manifestations are dry skin, erythema and self‐induced excoriations, commonly at the scalp, face, neck and flexural surfaces of the extremities.^2^ These allergic pruritic diseases – and AD in particular – occur due to excessive immune responses of the CD4 Th2 'phenotype'. These are usually due to genetic predisposition, but environmental factors also play a role. The activation of this immune pathway leads to IgE production and the classical clinical manifestations of hypersensitivity. The disease therefore requires long‐term treatment.^1^

Standard‐of‐care therapies include several possible lines of clinical intervention. Among these, the treatment of concurrent infections, the control of allergens and the use of anti‐histamines, glucocorticoids (topically or systemically) and, more recently, a Janus Kinase inhibitor, oclacitinib.^1^ In Japan, canine interferon gamma has been used in the treatment of AD. The goal of the therapy is to revert the Th1/Th2 immune disbalance that leads to excessive IgE responses.^3^, ^4^ Clinical efficacy was demonstrated in dog trials having antihistamine as the active control.^5^ Here, we report the use of recombinant human interferon‐alpha14 (rhIFNα−14) in the treatment of canine allergic dermatitis.^6^ Previous work has shown the molecule to interact with canine whole blood (unpublished data). A small‐scale trial was conducted to assess safety and initial efficacy of rhIFNα‐14.

## MATERIALS AND METHODS

This study was designed as an open uncontrolled trial. Eight dogs with chronic non‐seasonal AD were selected. Dogs were enrolled based on previously published inclusion criteria.^7^ Briefly, dogs had to have moderate to severe itching associated with allergy based on history, clinical signs and owner complaint. Dogs were otherwise healthy, were not in, and did not require, active treatment for other conditions; dogs were not receiving and had not received immunosuppressants, antibiotics or antihistamines for 8 weeks prior to the study. Inclusion to the study was also based on elimination of resembling non‐immune‐mediated pruritic dermatoses by clinical assessment only. The owners received explanations about the trial, and the study included those who accepted and completed the informed consent form. The study was performed under the license of the Committee for the Use of Animals in Research of Imunova Análises Biológicas, protocol 003.2018. The trial was conducted according to the relevant international guidelines in ethics in the use of animals in research.

All dogs were treated with the experimental compound. Recombinant human interferon‐alpha14 was produced in *E. coli* by Invigate GmbH, Jena, Germany and shown to be >98% pure by SDS‐PAGE and MS. The anti‐viral bioactivity was assessed by U‐CyTech Biosciences, Utrecht, The Netherlands and shown to be 1.8 × 10^8^ IU/mg. Lyophilized interferon was resuspended in 0.1% bovine serum albumin in saline and was frozen in aliquots until the time of use. Three animals received 10,000 IU/kg of rhIFNα−14 via subcutaneous injection. The protocol for parenteral treatment consisted of administration three times weekly for 4 weeks, then once weekly for another 4 weeks. Five animals received the formulation at the same dosage via oral administration, daily for 8 weeks.

'Within‐treatment' follow‐up was comprised of clinical assessment and efficacy outcomes (veterinarian and owner assessments). Each dog was evaluated for the presence or absence of papule, macula, pustules, dandruff, skin scabs, lichenification, nodules, tumours, hyperkeratosis, vesicles, hyperpigmentation, erythema and alopecia by the veterinarian once a week. Efficacy outcomes were based on the veterinary‐conducted version of the CADESI (canine AD extent and severity index) score used in previous trials. This was based on assigning scores (1 to 5) to the levels of pruritus, excoriation, erythema and alopecia. Scoring was performed according to a table that defined the scores based on the size of the lesion and its characteristics.^5^, ^7^ Owner assessment of the status of the dog was also collected during the clinical consultation. A visual analogue scale was used by owners for quantification of observed pruritus. A mark was made on a 15‐cm long line to indicate the degree of observed behaviour.^7^ This was then measured with a ruler and used for statistical analyses. Dogs had to have been in the study until at least week 3 to be included in the efficacy analysis (which excluded one dog from the evaluation).

Blood samples were collected for haematological analysis and for quantification of anti‐interferon antibodies. Samples were collected weekly whenever there was owner compliance. Therefore, sampling was not homogeneous between dogs throughout the test. Haematological analyses consisted of determination of blood cell counts, alkaline phosphatase, urea, creatinine, total protein, protein fractions and AST levels. This was performed at the Veterinary Hospital at the Universidade Federal do Paraná. For the determination of adverse events, deviations in blood parameters that occurred following drug administration were counted. ELISA for determination of canine anti‐rhIFNα14 antibodies was performed in house. Briefly, MaxiSorp plates (Nunc) were coated with 100 ng interferon/well. The plate was blocked with 1% casein in phosphate buffered saline (PBS). Control wells were coated with casein only. Plates were washed with PBS following each step. Serum was added in twofold dilutions for the determination of titres. HRP‐coupled anti‐dog IgG was used for detection of bound antibodies (Alpha Diagnostics, 1:10,000 dilution). The reaction was developed with TMB liquid substrate (Life Scientific). The absorbance of interferon‐coated wells was subtracted from the absorbance from control wells (coated with casein only) for the determination of specific anti‐interferon antibody titres.

Long‐term follow‐up efficacy assessment was performed with the best responders by communication with the owners at 5 months post‐trial. Owners were asked for the status of the dogs and the time to return of pruritus. There was no clinical assessment of the dogs during the follow‐up period.

Skin microbiota was assessed by next‐generation sequencing. Skin swabs were collected at treatment D0 and at the end of the trial for three dogs. DNA was extracted from swabs using the ZR Fecal DNA MiniPrep (Zymo Research). The variable V4 region of 16S rRNA was amplified using the universal primers 515F and 806R (Caporaso et al, 2011). PCR conditions were as follows: 94°C, 3 minutes; 18 cycles of 94°C, 45 seconds, 50°C, 30 seconds e 68°C, 60 seconds; followed by 72°C, 10 minutes. These amplicons were then sequenced (Illumina MiSeq). Sequencing reads were normalised at 8010 reads and analysed with the QIIME (Quantitative Insights into Microbial Ecology) platform (Caporaso et al, 2010, 2011). Sequences were classified into bacterial genera through the recognition of operational taxonomic units based on the homology at 97% of the sequences when compared to the SILVA 128 ribosomal sequence database (2017 release) (Yilmaz et al, 2013). Basic diversity was assessed using QIIME.

Statistical analysis was conducted using MiniTab 7 and GraphPad Prism 6. Owner pruritus score and veterinarian evaluation score percentual changes in relation to the start of the trial were assessed by Wilcoxon Signed Rank test. ELISA and microbiome results were interpreted with Mann‐Whitney tests. *p* was considered significant when <0.10.

## RESULTS

**FIGURE 1:**
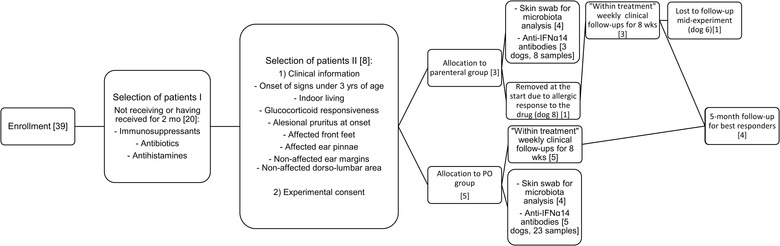
Experimental layout and number of animals [in brackets] in each stage. During the enrolment phase the number of candidate dogs was reduced as they fell into the exclusion criteria. Dog 8 was not considered in the efficacy analyses as it left the trial following a single dose of interferon

Three males and five females were enrolled in the trial. They were assigned into two groups, for parenteral (*n *= 3 dogs) or oral (*per os* [PO]) (*n = *5 dogs) routes of treatment. Experimental layout and the number of animals in each phase of the trial are shown in Figure 1.

### Adverse events

During the trial, severe adverse drug reactions were not observed. Although there were deviations from the reference parameters in blood tests, these were not correlated with any clinical manifestation (Table 1). The most severe reactions were observed in dogs that received the subcutaneous injections (Dogs 6, 7 and 8). Treatment of dog 6 was discontinued in the third week due to adverse effects including skin rashes and fever, vomiting and nausea. The owner of dog 8 stopped treatment after the first dose due to intense skin rashes.

**TABLE 1:** Adverse events: The number of blood samples was counted when one or more blood tests from the same animal showed deviations from standard parameters. Adverse events not related to the blood tests are also listed

Parameters	Parenteral injection (N: three dogs, five samples after the first dose*)	Oral administration(N: five dogs, 10 samples after the first dose*)
	Fraction of positive samples	
Albuminemia	1/1	7/8
Increased packed cell volume	0/3	4/9
Increased haemoglobin	1/3	6/9
Increased plasma proteins	2/3	1/10
Leucocytosis	1/3	1/8
Eosinophilia	0/3	4/8
Neutrophilia (segmented)	1/3	3/8
Lymphopenia	1/3	2/8
Monocytopenia	2/3	8/8
Vomiting	1/3	0/10
Skin rashes	2/3	0/10

* Not all samples were processed for all analyses due to small blood samples.

### Efficacy

**FIGURE 2:**
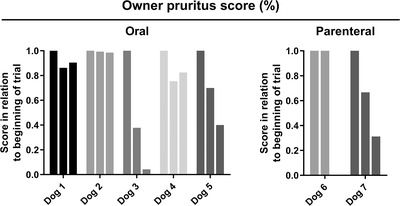
Subjective pruritus score by owners. Results were normalised in relation to the score of each dog at the beginning of the trial. There are 3 bars for each dog, indicating the beginning, the middle and the end of the trial. Dates for these periods are approximated between dogs, since many owners did not comply with the determined evaluation dates. Data are reported as fractions from the start of the trial for each dog. Dogs 4, 5 and 8 received the drug by the parenteral route. The third bar is missing from dog 4 as it left the trial by the 3rd week. Dog 8 is missing as it left the trial following the first dose

Efficacy results include both administration routes. Although this was not basis for enrolment of the dogs in the trial, patients shared a common history of non‐responsiveness to previous veterinary interventions. A subjective scoring system was used for quantifying the opinion of the owners regarding effectiveness of the treatment. The results of this subjective score are shown in Figure 2. By the end of the test, three dogs had greater than 60% reduction of pruritus, whereas two dogs had less than 20% reduction. Two dogs showed no reduction in pruritus according to the owners, and dog 8 was removed from the analysis as it showed allergic signs following the first administration of the drug. The aggregate result was of 42% reduction of pruritus by the end of the trial. For the oral route of treatment, there was a mean reduction of pruritus of 24.6% mid‐trial and of 17.5% by the end of the trial. Both mid‐trial (*p *= 0.062) and end‐of‐trial scores (*p *= 0.062) were reduced in relation to the start of the trial for orally treated animals and for the total scores (*p *= 0.047 and 0.031 for mid and end‐of‐trial, respectively). The parenteral route could not be analysed separately as the small number of cases prevents the use of the Wilcoxon Signed Rank Test. Nevertheless, of these ‘low‐responders’, the owner of dog 4 reported that it stopped waking up by the end of the protocol, even though the total score was not greatly reduced. In addition, three owners (of dogs 3, 4 and 7) reported increased feed consumption by the end of the trial (and these dogs effectively gained weight).

**FIGURE 3:**
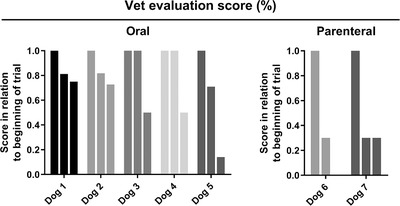
Veterinarian evaluation scores. (a) Data from the summation of scores for excoriation, erythema and alopecia. There are three bars for each dog, indicating the beginning, the middle and the end of the trial. Dates for these periods are approximated between dogs, since many owners did not comply with the determined evaluation dates. Data are reported as fractions from the start of the trial for each dog. Dogs 4, 5 and 8 received the drug by the parenteral route. The third bar is missing from dog 4 as it left the trial by the 3rd week. Dog 8 is missing as it left the trial following the first dose

Every week, on return for veterinarian evaluation, the investigator evaluated skin lesions. There was an overall reduction of the lesions of 49% by the end of the experiment. Dogs 3, 4, 6 and 7 had a 50% or greater reduction of the lesions by the termination of the trial (Figure 3). Mid‐trial scores (*p *= 0.062) and final scores (*p *= 0.031) were reduced in relation to the start of the experiment. When the oral route of administration was considered in isolation, mid‐trial results were not different from the start of the protocol (*p *= 0.25) but were significantly lower by the end of the experiment (*p *= 0.062).

### Anti‐interferon antibodies

**FIGURE 4:**
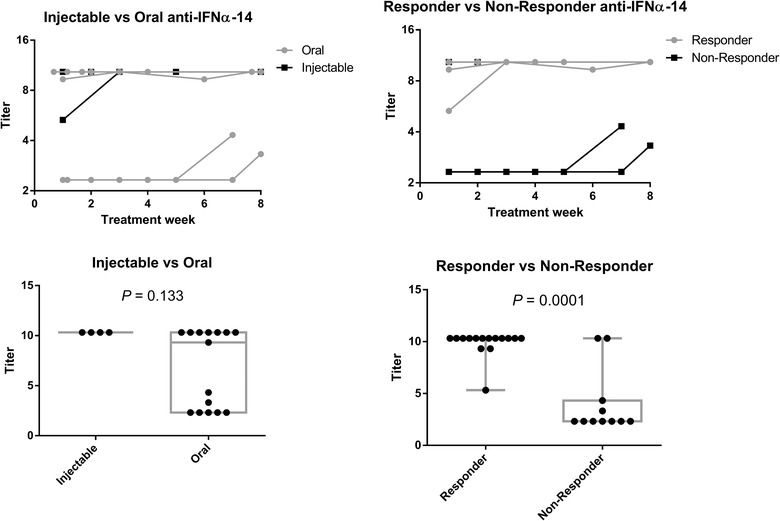
ELISA for detection of canine anti‐rhIFNα14 antibodies. Top: antibody titers by treatment week. Each line represents an animal. No statistical analysis was performed because there are missing data. Below: antibody titres were grouped for statistical comparisons. For the comparison between injectable and oral treatments, titres at D0 were removed from the analysis as the goal was to assess the induction of anti‐antibodies following treatment. Statistical analysis by two‐tailed Mann‐Whitney test. In the timeline analysis, geometric means are shown. In the aggregated analysis, each dot is a blood collection point

Anti‐rhIFNα−14 antibodies were measured in the serum of all dogs. Due to the low number of animals, statistical comparisons throughout time are hampered, but anti‐interferon antibodies were lower in responders (dogs 3, 4, 5 and 7 were classified into this group) in relation to non‐responders (dogs 1, 2, 6 and 8). This is confirmed when all sera were pooled for group comparison. No significance was found in the difference between injectable dosing and oral administration (Figure 4).

### Microbiota

Skin microbiota of three responders was assessed at treatment D0 and at the end of the protocol (dogs 3, 4 and 7). The microbiota of Dog 2 was also assessed at D0, but its analysis at the end of the protocol failed to generate data.

**FIGURE 5:**
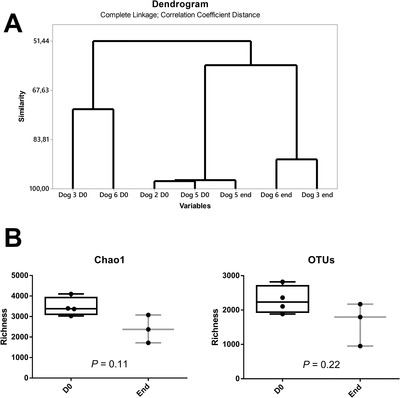
Analysis of the skin microbiota. (a) Overall similarity of the skin microbiota between dogs at the start of the trial and at the end. Dog 5 showed very distinctive trends regarding its microbial skin composition. It finished the trial with over 90% of the skin microbiota composed of *Staphylococcus*. (b) Microbiota richness at the start of the trial versus the end of the test. Chao1 and OTU richness evaluations are shown. Statistical analysis by Mann‐Whitney test. ‘D0’, beginning of the trial with interferon. ‘End’, finalization of the trial

Overall analysis of the skin microbiota demonstrated that treatment induced changes by the end of the protocol (Figure 5). Several differences were found between the beginning and the end of the protocols. In all three animals there was a significant increase in the number of Firmicutes and a reduction in Actinobacteria. The most common bacterial genera (top 15%) were *Staphylococcus, Streptococcus, Lactobacillus, Corynebacterium, Pseudomonas*. Of these, dogs 3 and 4 (both received interferon orally), which were the most similar, had a relatively low percentage of *Staphylococcus* (ca. 5%). Treatment had differing effects on this bacterial population in dogs 3 and 4: it decreased (0.87‐fold) *Staphylococcus* following treatment in dog 3 but increased it in 4 (4.4‐fold). Dog 7 (parenteral route of administration), in which *Staphylococcus* represented 24% of the bacteria at the start of the test, also had a marked increase in the relative number of *Staphylococcus* following treatment (3.8‐fold). These bacteria reached over 90% of the skin bacterial population of dog 7 by the end of the protocol, although it did not have any signs of dermatitis at the time. Dogs 3 and 4 also shared an increase in the relative abundance of *Pseudomonas* and *Lactobacillus*, a trend which was again opposite in dog 7. In common, dogs 3, 4 and 7 shared a decrease in the percentage of *Corynebacterium* and *Actinomyces* following treatment.

### Long‐term follow‐up

Follow‐up data were obtained from the best responders to interferon treatment (dogs 3, 5, 6 and 7). Dog 3 remained free from any sign of allergic pruritus for at least 5 weeks following the end of the trial. Dog 7 remained free from intense pruritus for 45 days. By 4 months of the trial, pruritus on the face and extremities had returned, but not yet to the same level of the beginning of the trial. Dog 4 and 5 showed return of pruritus and skin lesions. The owner of dog 4 reported slow return of the signs starting from 3 to 4 weeks from the end of the test. By 3 months, the original lesions and pruritus were similar to the start of the trial. Dog 5 showed a fast return to the original condition, which happened by 2 weeks of the end of the trial.

## DISCUSSION

Allergic pruritic diseases of the dog skin are characterised by a deviation of the immune responses towards a T helper type 2 phenotype (Th2). There is increased production of cytokines by Th2 polarized cells which leads to the intense pruritus that is common to these conditions, regardless of the causative allergen.^8^ Therefore, systemic treatment options include either glucocorticoids or cyclosporin, anti‐histamines or, more recently, a small molecule inhibitor and an antibody that block pruritus via the IL‐31 signalling pathway.^5^, ^9^, ^10^, ^11^ Nevertheless, immunosuppressants often lead to severe adverse effects, especially because therapy is lifelong. Anti‐histamines are only useful as supplementary treatments.^12^ The novel molecules, on the other hand, can be prohibitively costly, especially in poorer countries, that import the molecules (anecdotal evidence). Since the prevalence of these diseases is high (in the order of 10% of the canine population), novel drugs may prove useful.

Interferons have been trialled and used in the treatment of human AD for their role in reverting the Th2 disbalance, for effects on keratinocytes and for reducing secondary skin infections.^13^, ^14^ In Japan, canine AD has been treated with canine recombinant interferon gamma, made available in the country by Toray Industries.^3^ This drug shows good efficacy, reducing pruritus, excoriation, erythema and alopecia by 57–79%.^4^, ^5^ Using a similar trial approach as has been reported by Japanese research groups, we show here that recombinant human interferon alpha 14 (rhIFNα−14) induced a mean decrease in pruritus of 42%, considering owner evaluation of pruritus and of 49%, considering vet‐assigned lesion scores.

The dosage scheme followed by our group was similar to those previously reported for IFNγ.^4^, ^5^ However, injectable interferon induced prominent allergic reactions in two of the three dogs that received it. Some dogs showed significant levels of specific antibodies even before the start of the trial, and we could not find an association between anti‐interferon antibodies and clinical efficacy. Total levels of anti‐drug antibodies are not necessarily associated with loss of function, since most immunoglobulins are not neutralizing, perhaps justifying why even 'responder' dogs had high antibody titres.^15^ Pre‐existing antibodies that cross‐react with biological drugs are not rare, and they usually have little impact in treatment outcomes, as was found in the present study.^16^ High prevalence of pre‐existing cross‐reactive antibodies in the dogs may be due to the chronic immune activation that drives allergic dermatitis. It is known that AD patients are in higher risk of other inflammatory‐driven diseases, for instance.^17^

Orally administered interferon showed some efficacy in three of five dogs that received it, with no adverse events. Oral interferon has been tested for the treatment of other conditions in dogs and cats.^18^, ^19^, ^20^ However, its use for AD in dogs is rather novel.^4^, ^5^, ^21^ In contrast, adverse events were considerable in two of the three dogs that received parenteral interferon. These dogs showed classical signs of hypersensitivity against the recombinant protein, with skin rashes and vomiting.^22^ Such adverse events due to parenteral administration of biologicals are not rare and may derive from classical hypersensitivity mediated by IgE – especially since it was a human recombinant protein – but also from intense immune activity following the administration of an exogenous cytokine.^23^ Since there were few dogs in the ‘parenteral arm’ of the study, these results do not necessarily preclude using this route in future trials.

Failures in treatment efficacy may be due to the simplified selection of enrolled dogs. Pruritus in dogs is often derived from immune‐mediated processes, and the selection criteria attempted to narrow towards patients that with these pathological characteristics. However, itch may also arise from neurological or psychological conditions, which cannot be excluded as causes in our enlisted dogs.^24^ It is expected that rhIFNα−14 would not benefit such patients, since the underlying pathogenic mechanisms are vastly different.

The skin microbiota is altered in canine AD. However, we could not observe the apparent correlation of *Staphylococcus* spp. with clinical outcomes reported by others.^25^, ^26^, ^27^ Our results showed varying effects of treatment over *Staphylococcus* spp. prevalence on the skin of dogs. Class I interferons – such as IFNα – have a dual role on *Staphylococcus* spp. colonization of the skin. These cytokines may enhance skin immune responses against *S. aureus*, influencing bacterial loads.^28^ Opposingly, chronic exposure to interferons disrupts the skin barrier, increasing staphylococcal colonization.^29^, ^30^ These divergent effects of interferons may account for the different outcomes regarding skin *Staphylococcus* spp. abundance in our trial. The connection of other bacterial species with AD is less established. As an example, some reports indicate higher *Corynebacterium* spp. percentages in the skin of healthy dogs,^26^ whereas opposing results can also be found, and therefore it is difficult to interpret the results found here regarding this bacterial genera.^25^, ^27^

The skin microbiota varies greatly with each individual, with the body site to be considered, time of sampling and with cohabitation.^26^, ^31^, ^32^ Therefore, it is difficult to compare microbiome results between dogs due to natural individual variations and to different sites of sampling, since swabs were taken from lesions, which varied in location between dogs. The long interval between the beginning and the end of the trial and the low number of dogs that were tested also represent hurdles in the interpretation of these data. Nevertheless, it is interesting to observe that microbiotas on day 0 grouped together in the dendrogram analysis, apart from those from the end of the test. Therefore, there is an indication of effect of the treatment over the microbiota.

In conclusion, administration of rhIFNα−14 induced varied outcomes in the treatment of allergic pruritic conditions in dogs. The results indicate that novel trials should concentrate in the oral route of administration, as the adverse effects (rashes and vomiting) seen in dogs that were injected suggest that parenteral administration is not likely to be safe.

## AUTHOR CONTRIBUTIONS

Breno C. B. Beirão and William H. Stimson devised the trial. Breno C. B. Beirão, Aline C. Taraciuk, Max Ingberman, Carolina Trentin and Luiz F. Caron ran the clinical trials. Breno C. B. Beirão, Aline C. Taraciuk and Chris McKenzie ran laboratory tests. Breno C. B. Beirão wrote the first draft of the article. All authors contributed to its revision and improvement. Breno C. B. Beirão is responsible for the overall content as guarantor.

## References

[1] Olivry T , DeBoer DJ , Favrot C , Jackson HA , Mueller RS , Nuttall T , et al. Treatment of canine atopic dermatitis: 2015 updated guidelines from the International Committee on Allergic Diseases of Animals (ICADA). BMC Vet Res. 2015;11:210. 26276051 10.1186/s12917-015-0514-6 PMC4537558

[2] Hillier A , Griffin CE . The ACVD task force on canine atopic dermatitis (I): incidence and prevalence. The ACVD task force on canine atopic dermatitis (I): incidence and prevalence. Vet Immunol Immunopathol. 2001;81:147–51. 11553375 10.1016/s0165-2427(01)00296-3

[3] Day MJ . Clinical immunology of the dog and cat. 2n ed. Boca Raton, FL: CRC Press; 2011.

[4] Yasukawa K , Saito S , Kubo T , Shibasaki Y , Yamaoka K , Hachimura H , et al. Low‐dose recombinant canine interferon‐γ for treatment of canine atopic dermatitis: An open randomized comparative trial of two doses. Vet Dermatol. 2010;21:42–9. 19706009 10.1111/j.1365-3164.2009.00764.x

[5] Iwasaki T , Hasegawa A . A randomized comparative clinical trial of recombinant canine interferon‐γ (KT‐100) in atopic dogs using antihistamine as control. Vet Dermatol. 2006;17:195–200. 16674735 10.1111/j.1365-3164.2006.00519.x

[6] Stimson W . Compositions and methods relating to the treatment of diseases. United States Patent 10487127; 2016; https://www.freepatentsonline.com/10487127.html.

[7] Cosgrove SB , Wren JA , Cleaver DM , Martin DD , Walsh KF , Harfst JA , et al. Efficacy and safety of oclacitinib for the control of pruritus and associated skin lesions in dogs with canine allergic dermatitis. Vet Dermatol. 2013;24:479. 23829933 10.1111/vde.12047 PMC4282347

[8] McCandless EE , Rugg CA , Fici GJ , Messamore JE , Aleo MM , Gonzales AJ . Allergen‐induced production of IL‐31 by canine Th2 cells and identification of immune, skin, and neuronal target cells. Vet Immunol Immunopathol. 2014;157:42–8. 24321252 10.1016/j.vetimm.2013.10.017

[9] Olivry T , Sousa CA . The ACVD task force on canine atopic dermatitis (XX): glucocorticoid pharmacotherapy. Vet Immunol Immunopathol. 2001;81:317–22. 11553394 10.1016/s0165-2427(01)00314-2

[10] Gonzales AJ , Bowman JW , Fici GJ , Zhang M , Mann DW , Mitton‐Fry M . Oclacitinib (APOQUEL®) is a novel Janus kinase inhibitor with activity against cytokines involved in allergy. J Vet Pharmacol Ther. 2014;37:317–24. 24495176 10.1111/jvp.12101 PMC4265276

[11] Silva MABM da . Avaliação do uso de lokivetmab (Cytopoint) na dermatite atópica canina. Lisboa, Portugal: Universidade de Lisboa, Faculdade de Medicina Veterinária; 2019.

[12] Olivry T , Mueller RS . Dermatitis ITF on CA.: evidence‐based veterinary dermatology: a systematic review of the pharmacotherapy of canine atopic dermatitis. Vet Dermatol. 2003;14:121–46. 12791047 10.1046/j.1365-3164.2003.00335.x

[13] Brar K , Leung DYM . Recent considerations in the use of recombinant interferon gamma for biological therapy of atopic dermatitis. Expert Opin Biol Ther. 2016;16:507–14. 26694988 10.1517/14712598.2016.1135898 PMC4985031

[14] Torrelo A , Harto A , Sendagorta E , Czarnetzki BM , Ledo A . Interferon‐alpha therapy in atopic dermatitis. Acta Derm Venereol. 1992;72:370–2. 1361287

[15] Steis RG , Smith JW , Urba WJ , Clark JW , Itri LM , Evans LM et al. Resistance to recombinant interferon alfa‐2a in hairy‐cell leukemia associated with neutralizing anti‐interferon antibodies. N Engl J Med. 1988;318:1409–13. 3367950 10.1056/NEJM198806023182201

[16] van Schie KA , Wolbink G‐J , Rispens T . Cross‐reactive and pre‐existing antibodies to therapeutic antibodies–Effects on treatment and immunogenicity. MAbs. 2015;7:662–71. 25962087 10.1080/19420862.2015.1048411 PMC4623040

[17] Schmitt J , Schwarz K , Baurecht H , Hotze M , Fölster‐Holst R , Rodríguez E , et al. Atopic dermatitis is associated with an increased risk for rheumatoid arthritis and inflammatory bowel disease, and a decreased risk for type 1 diabetes. J Allergy Clin Immunol. 2016;137:130–36. 26253344 10.1016/j.jaci.2015.06.029

[18] Gilger BC , Rose PD , Davidson MG , Roberts SM , Miller T . Low‐dose oral administration of interferon‐alpha for the treatment of immune‐mediated keratoconjunctivitis sicca in dogs. J Interf cytokine Res. 2004;19:901–5. 10.1089/107999099313433 10476936

[19] Pedretti E , Passeri B , Amadori M , Isola P , Di Pede P , Telera A . et al. Low‐dose interferon‐α treatment for feline immunodeficiency virus infection. Vet Immunol Immunopathol. 2006;109:245–54. 16169599 10.1016/j.vetimm.2005.08.020

[20] Siebeck N , Hurley DJ , Garcia M , Greene CE , Köstlin RG , Moore PA , et al. Effects of human recombinant alpha‐2b interferon and feline recombinant omega interferon on in vitro replication of feline herpesvirus‐1. Am J Vet Res. 2008;67:1406–11. 10.2460/ajvr.67.8.1406 16881854

[21] Litzlbauer P , Weber K , Mueller RS . Oral and subcutaneous therapy of canine atopic dermatitis with recombinant feline interferon omega. Cytokine. 2014;66:54–9. 24548425 10.1016/j.cyto.2013.12.001

[22] Voie KL , Campbell KL , Lavergne SN . Drug hypersensitivity reactions targeting the skin in dogs and cats. J Vet Intern Med. 2012;26:863–74. 22519673 10.1111/j.1939-1676.2012.00927.x

[23] Corominas M , Gastaminza G , Lobera T . Hypersensitivity reactions to biological drugs. J Investig Allergol Clin Immunol. 2014;24:212–25. 25219103

[24] Grundmann S , Ständer S . Chronic pruritus: clinics and treatment. Ann Dermatol. 2011;23:1–11. 21738356 10.5021/ad.2011.23.1.1 PMC3119985

[25] Pierezan F , Olivry T , Paps JS , Lawhon SD , Wu J , Steiner JM . et al. The skin microbiome in allergen‐induced canine atopic dermatitis. Vet Dermatol. 2016;27:332. 27485242 10.1111/vde.12366

[26] Chermprapai S , Ederveen THA , Broere F , Broens EM , Schlotter YM , van Schalkwijk S , et al. The bacterial and fungal microbiome of the skin of healthy dogs and dogs with atopic dermatitis and the impact of topical antimicrobial therapy, an exploratory study. Vet Microbiol. 2019;229:90–9. 30642603 10.1016/j.vetmic.2018.12.022

[27] Bradley CW , Morris DO , Rankin SC , Cain CL , Misic AM , Houser T , et al. Longitudinal evaluation of the skin microbiome and association with microenvironment and treatment in canine atopic dermatitis. J Invest Dermatol. 2016;136:1182–90. 26854488 10.1016/j.jid.2016.01.023 PMC4877200

[28] Klopsfenstein N , Brandt S , Castellanos S , Serezani C . SOCS‐1 inhibition of type I interferon limits Staphylococcus aureus skin host defense. bioRxiv. 2020. 10.1101/2020.09.28.317107. PMC7984627 33690673

[29] Sirobhushanam S , Parsa N , Sarkar MK , Gudjonsson JE , Kahlenberg JM . Staphylococcus aureus colonization is influenced by cutaneous interferon production. Am Assoc Immnol. 2018;200:117.9

[30] Sirobhushanam S , Parsa N , Reed TJ , Berthier CC , Sarkar MK , Hile GA , et al. Staphylococcus aureus colonization is increased on lupus skin lesions and is promoted by IFN‐mediated barrier disruption. J Invest Dermatol. 2020;140:1066–74.e4. 31877319 10.1016/j.jid.2019.11.016 PMC7183889

[31] Torres S , Clayton JB , Danzeisen JL , Ward T , Huang H , Knights D , et al. Diverse bacterial communities exist on canine skin and are impacted by cohabitation and time. PeerJ. 2017;5:e3075. 28289569 10.7717/peerj.3075 PMC5346284

[32] Cuscó A , Sánchez A , Altet L , Ferrer L , Francino O . Individual signatures define canine skin microbiota composition and variability. Front Vet Sci. 2017;4:6. 28220148 10.3389/fvets.2017.00006 PMC5292769

